# Synthesis of 1,3,4-Thiadiazole Derivatives and Their Anticancer Evaluation

**DOI:** 10.3390/ijms242417476

**Published:** 2023-12-14

**Authors:** Camelia Elena Stecoza, George Mihai Nitulescu, Constantin Draghici, Miron Teodor Caproiu, Anamaria Hanganu, Octavian Tudorel Olaru, Dragos Paul Mihai, Marinela Bostan, Mirela Mihaila

**Affiliations:** 1 Faculty of Pharmacy, “Carol Davila” University of Medicine and Pharmacy, 6 Traian Vuia Street, 020956 Bucharest, Romania; camelia.stecoza@umfcd.ro (C.E.S.); octavian.olaru@umfcd.ro (O.T.O.); dragos_mihai@umfcd.ro (D.P.M.); 2 “Costin D. Neniţescu” Institute of Organic and Supramolecular Chemistry, Romanian Academy, 202B Splaiul Independenţei, 060023 Bucharest, Romaniaanamaria.hanganu@unibuc.ro (A.H.); 3 Stefan S. Nicolau Institute of Virology, 285 Mihai Bravu Street, 030304 Bucharest, Romania; marinela.bostan@virology.ro (M.B.); mirela.mihaila@virology.ro (M.M.)

**Keywords:** thiadiazole derivatives, LoVo cells, MCF-7 cells, *Daphnia magna*, PASS prediction, cell cycle analysis, induction of apoptosis, 2-amino-1,3,4-thiadiazole

## Abstract

Thiadiazole derivatives have garnered significant attention in the field of medicinal chemistry due to their diverse pharmacological activities, including anticancer properties. This article presents the synthesis of a series of thiadiazole derivatives and investigates their chemical characterization and potential anticancer effects on various cell lines. The results of the nuclear magnetic resonance (NMR) analyses confirmed the successful formation of the target compounds. The anticancer potential was evaluated through in silico and in vitro cell-based assays using LoVo and MCF-7 cancer lines. The assays included cell viability, proliferation, apoptosis, and cell cycle analysis to assess the compounds’ effects on cancer cell growth and survival. *Daphnia magna* was used as an invertebrate model for the toxicity evaluation of the compounds. The results revealed promising anticancer activity for several of the synthesized derivatives, suggesting their potential as lead compounds for further drug development. The novel compound **2g**, 5-[2-(benzenesulfonylmethyl)phenyl]-1,3,4-thiadiazol-2-amine, demonstrated good anti-proliferative effects, exhibiting an IC_50_ value of 2.44 µM against LoVo and 23.29 µM against MCF-7 after a 48-h incubation and little toxic effects in the *Daphnia* test.

## 1. Introduction

Cancer, the uncontrolled division of cells, is one of the most challenging problems of worldwide public health. The global cancer prevalence is expected to be 28.4 million cases in 2040, a 47% rise from 2020 [1]. Although, in recent years, anticancer therapy has progressed significantly, there is an urgent necessity for the development of new drugs to kill cancer cells, as well as improved anticancer therapeutic strategies.

Nowadays, five-membered nitrogen- and oxygen- or sulfur-containing heterocycles have become an area of increasing interest in the context of designing new anticancer molecules [2,3]. Among these, thiadiazoles received particular interest. There are many research reports on the antitumor therapeutic potential of thiadiazole derivatives, especially of the thiadiazole molecules containing a simple unfused 2,5-disubstituted ring. Some compounds have been shown to have promising anticancer activity, exceeding the reference drugs in tests. The therapeutic potential of thiadiazole is influenced by the fact that its heterocyclic ring is a bioisoster of pyrimidine, which is the backbone of the structures of three nucleobases. Therefore, thiadiazoles have the ability to interfere with processes relate to DNA replication [4,5,6].

**Figure 1:**
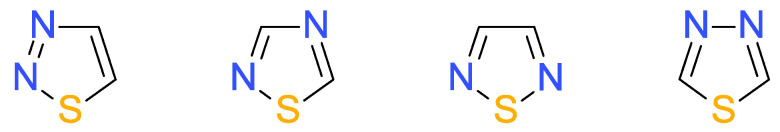
Structural formula of isomeric 1,2,3-, 1,2,4-, 1,2,5 and 1,3,4-thiadiazole.

The biological activity exhibited by the thiadiazoles is owed to their potential to bind to various targets through a broad range of interactions such as hydrogen bonding, van der Waals and hydrophobic forces and metallic coordination bonds [3,7,8]. Depending on the position of the nitrogen and sulfur atoms, the heterocycle may occur in one of the following four different isomeric forms: 1,2,3-, 1,2,4-, 1,2,5 and 1,3,4-thiadiazole [2,6], as presented in Figure 1.

Amongst them, 1,3,4-thiadiazoles are the most promising group in terms of antitumoral potential. Their pharmacological properties are related to the high aromatic character of the ring that imparts significant in vivo stability to this five-membered ring system, accompanied by minimal toxicity [9,10]. Due to their mesoionic nature, 1,3,4-thiadiazoles are capable of easily crossing cellular membranes and interact strongly with biological targets, such as proteins and DNA. Despite their internal charges, the mesoionic compounds are neutral and efficiently cross membranes, leading to good oral absorption and resulting in good bioavailability [2,4,5,11,12,13].

The 2-amino-1,3,4-thiadiazole structure has emerged as a promising foundation for the development of anticancer agents, which are compounds with antitumor properties in various in vitro and in vivo models and target a diverse range of molecular pathways. It was observed that the anticancer effect is usually enhanced by introducing an aromatic ring at the vacant 5th position of the 1,3,4-thiadiazole core. The efficacy of these compounds against cancer cells is influenced by the specific position and chemical nature of substituents on the aromatic ring, as well as the type of substituent attached to the amino group [14,15,16].

**Figure 2:**
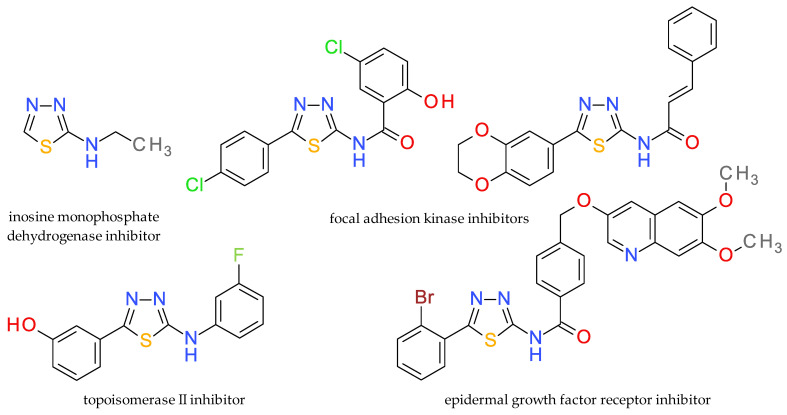
Structural formula of anticancer derivatives based on the 1,3,4-thiadiazole-2-amine scaffold.

Some derivatives of 1,3,4-thiadiazole-2-amine have gained attention as inhibitors of inosine monophosphate dehydrogenase (IMPDH), an enzyme involved in the de novo synthesis of guanosine nucleotides and which is crucial for the proliferation of cancer cells and the replication of certain viruses [4]. A series of amide derivatives of 5-aryl substituted 1,3,4-thiadiazole-2-amine were developed as micromolar inhibitors of the focal adhesion kinase (FAK) by targeting the ATP-binding pocket [17]. The antitumor capacity of 1,3,4-thiadiazole-2-amine derivatives is also related to their potential to inhibit topoisomerase II [18], glutaminase [19], histone deacetylase [20], Abl kinase [21] or the human epidermal growth factor receptor [22]. A series of representative 1,3,4-thiadiazole-2-amine derivatives are presented in Figure 2.

Considering the information provided above, we have hypothesized that exploring cytotoxic compounds within the category of 1,3,4-thiadiazole derivatives holds significant promise as an avenue of research. Consequently, we present our work on the synthesis, characterization, and biological assessment of a series of 1,3,4-thiadiazole derivatives which share a central 5-phenyl-1,3,4-thiadiazol-2-amine scaffold. Among these, three compounds, **2f**,**g** and **3c**, are new derivatives. In order to correlate the antitumor activity to a structure variation, we synthetized compounds bearing different substituents on the 5-phenyl fragment (compounds **2b**–**e**) and also compounds with different substituents attached to the amino group (compounds **3a**–**b**). The newly developed compounds, identified as **2f**,**g** and **3c**, contain a (phenylsulfanylmethyl)phenyl moiety, drawing inspiration from earlier anticancer studies within a category of oxadiazole derivatives characterized by the aforementioned structural motif [23]. These oxadiazole compounds have exhibited robust anticancer activity, as illustrated by 2-[2-(phenylsulfanylmethyl)phenyl]-5-(4-pyridyl)-1,3,4-oxadiazole, which demonstrated anti-proliferative effects and induced apoptosis in colon and breast adenocarcinoma cells.

Through a comparative analysis of the effects of the compounds on the growth and survival of cancer cells, we aimed to better understand the structure–activity relationships and the potential mechanistic pathways underlying the action of the novel compounds.

## 2. Results

### 2.1. Synthesis Procedures

A series of 10 derivatives of 1,3,4-thiadiazole-2-amine were synthesized in order to evaluate their effect on cancer cells. Compounds **2f**,**g**, and **3c** are reported here for the first time. All the compounds were analyzed by NMR and IR spectra, as well as by an elemental analysis, to demonstrate their structure and chemical purity.

### 2.2. Anti-Cancer Evaluation

Tumor cells often exhibit dysregulation in the normal progression of the cell cycle, leading to an abnormal and uncontrolled proliferation, which contributes to the progression of the tumor. Furthermore, tumor cells typically have an impaired ability to undergo apoptosis, a natural process of programmed cell death. In the context of cytostatic treatment, the primary objective is to induce apoptosis in tumor cells and to hinder their cell cycle progression to impede further proliferation [24].

#### 2.2.1. Effects on Cell Viability

The MTS assay was employed to assess the cell viability following the treatment with the 1,3,4-thiadiazole derivatives **2a**–**g** and **3a**–**c**, using Cis-Pt and DOX as reference drugs. To determine the optimal working concentration, dose-effect curves were made for each compound in the range of concentrations between 6.25 and 400 μM.

**Figure 3:**
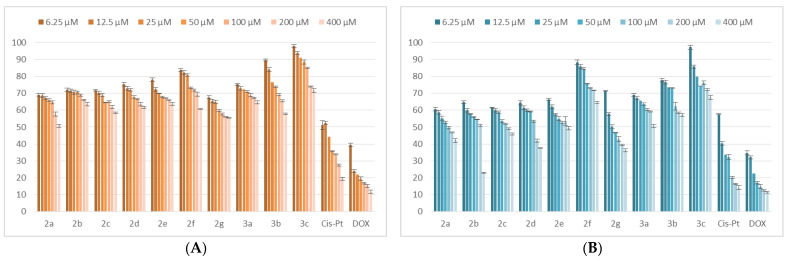
The tested compound effects on HUVEC normal cells’ viability. The cells were treated with the compounds and reference drugs Cis-Pt and DOX in the range of concentrations 6.25–400 μM for 24 h (**A**) and 48 h (**B**). Untreated cells were considered to have 100% viability.

The viability assay demonstrated that the analyzed compounds affect the viability of normal cells of HUVEC only, when high concentrations (100–400 μM) are used. The reduction of the viability was higher after 48 h, compared with the cells exposed for 24 h. The results in HUVEC cells are presented in Figure 3.

**Figure 4:**
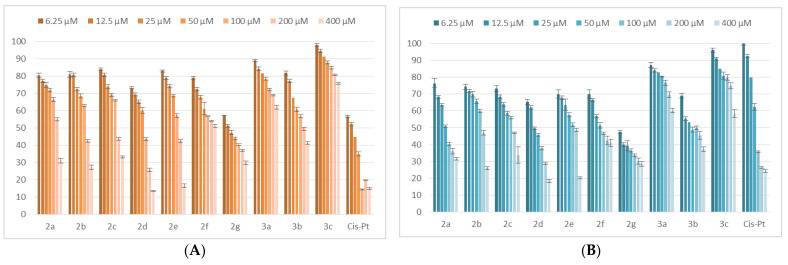
The tested compounds effects on LoVo tumoral cells’ viability. The cells were treated with the compounds and reference drug Cis-Pt in the range of concentrations 6.25–400 μM for 24 h (**A**) and 48 h (**B**). Untreated cells were considered to have 100% viability.

The analysis of the viability data obtained after treating the LoVo tumor line with the synthesized compounds shows that **2g** significantly reduces the cell viability even at small concentrations. Of the other analyzed compounds, **2a**–**e** and **3b** reduce the viability of LoVo tumor cells below 50% when applied for 24 h in concentrations of 200 μM. The profile of the effects on cell viability was similar when the treatments were carried out for 48 h, with a higher potency. With the exception of **3a**,**c**, all the tested thiadiazoles reduced the viability below 50% at 200 μM. The results in LoVo cells are presented in Figure 4.

**Figure 5:**
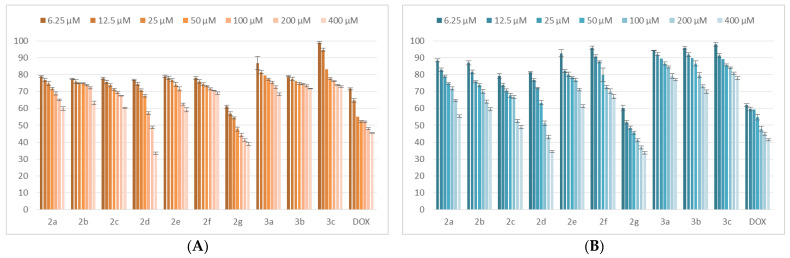
The tested compound effects on MCF-7 tumoral cells’ viability. The cells were treated with the compounds and reference drug DOX in the range of concentrations 6.25–400 μM for 24 h (**A**) and 48 h (**B**). Untreated cells were considered to have 100% viability.

The examination of viability data derived from the treatment of the MCF-7 tumor cell line with the synthesized compounds reveals that **2d**,**g** produced a significant reduction in cell viability after 24 h and 48 h. Compound **2c** induced a decrease in the viability to less than 50% when administered for a 48-h period, but not in the 24-h experiment. All the other tested thiadiazoles did not reduce the cell viability under the 50% value. The results pertaining to MCF-7 cells are depicted in Figure 5.

In order to better evaluate the compounds’ impact on the cell’s viability, the half-maximal inhibitory concentration (IC_50_) was calculated as a measure of the concentration that is necessary to inhibit cell growth or proliferation by 50%. The IC_50_ values were calculated only for the compound that produces an inhibition over 50%. The results are presented in Table 1 and the corresponding 95% confidence intervals are presented in the Supplementary Materials, Table S1.

The IC_50_ values indicates that all the tested thiadiazoles are more active on the LoVo cells than on MCF-7 cells. When compared to the effects on HUVEC cells, the compound’s inhibition on LoVo cells is several folds higher, with the exception of compound **2b**. Of particular note is compound **2g**, which stands out as the most potent and selective inhibitor, offering the best overall inhibitory effect. This compound also produced the best effect on MCF-7 cells in the 48-h experiment, but its selectivity is reduced, as it also has a significant effect on the HUVEC cells.

#### 2.2.2. Apoptosis Analysis

Two reference concentrations, 5 μM and 50 μM, and two distinct treatment durations, 24 and 48 h, were employed to assess the influence of the test compounds on apoptosis in both normal and tumor cells. Cis-Pt was used as a positive control for LoVo cells, while DOX was used in MCF-7 cells. HUVEC cells were exposed to both drugs. The results are reported as total apoptosis percentages (%) and are presented in Table 2.

In general, all of the tested 1,3,4-thiadiazole derivatives increased the proportion of apoptotic cells to be as high as 4.65 folds higher than the untreated cells. On average, the effect is higher after 48 h as compared to the 24 h exposure, and higher on MCF-7 cells compared to LoVo or HUVEC cells.

#### 2.2.3. The Effect on Cell Cycle Phases

The cell cycle analysis using flow cytometry evaluated the compound’s influence on cellular phase transitions. By comparing the relative proportions of cells in each phase, it was possible to determine the cell cycle dynamics, identify potential disruptions or abnormalities in cell division, and study the effects on cell proliferation.

**Figure 6:**
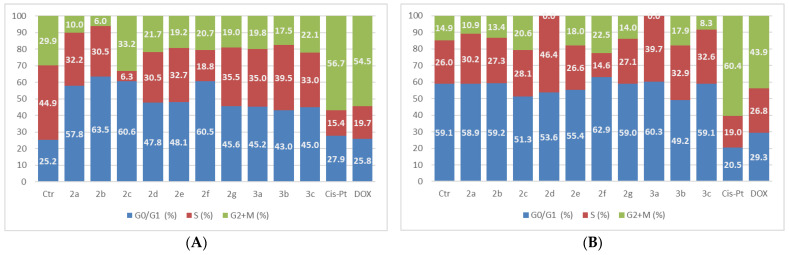
The tested compounds’ effects on cell cycle in HUVEC normal cells. The cells were treated with the compounds and reference drugs Cis-Pt and DOX at 5 µM for 24 h (**A**) and 48 h (**B**).

The results on HUVEC cells for the 5 µM dose treatment are presented in Figure 6.

The treatment of HUVEC cells with the analyzed compounds led to an increase in the G0/G1 phase after 24 h of exposure at 5 µM. The analysis of the cell cycle phases after 48 h of treatment did not record significant changes in the distribution of the cell cycle phases compared to untreated cells, with the exception of compound **2d**.

**Figure 7:**
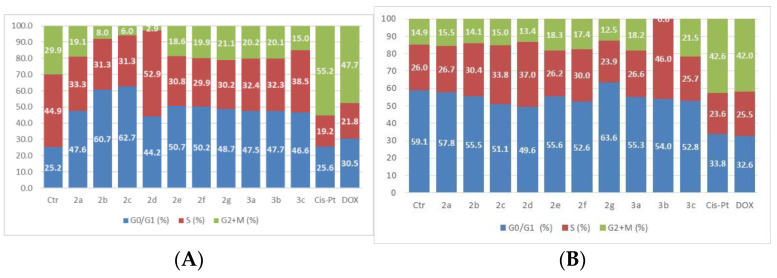
The tested compounds’ effects on cell cycle in HUVEC normal cells. The cells were treated with the compounds and reference drugs Cis-Pt and DOX at 50 µM for 24 h (**A**) and 48 h (**B**).

The results of the compounds on HUVEC cells for the 50 µM dose are presented in Figure 7.

The treatment of HUVEC cells with the analyzed compounds led to an increase in the G0/G1 phase after 24 h of exposure at 50 µM, with a corresponding decrease in the S phase and G2 + M. The assessment of cell cycle phases following a 48-h treatment revealed no notable changes in the distribution of these phases when compared to untreated cells, except in the case of compound **2b**.

**Figure 8:**
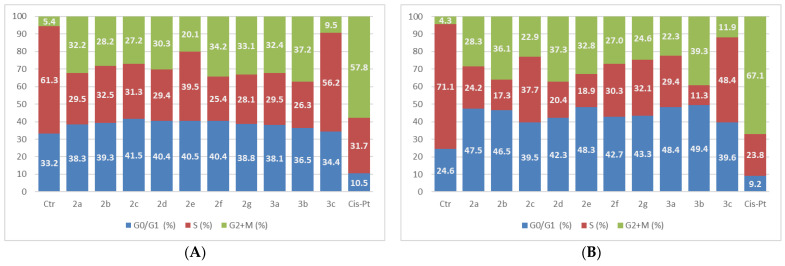
The tested compounds effects on cell cycle in LoVo cells. The cells were treated with the compounds and reference drug Cis-Pt at 5 µM for 24 h (**A**) and 48 h (**B**).

The results on LoVo cells for the 5 µM dose treatment are presented in Figure 8.

The compounds’ assay on LoVo cells determined different results after 48 h of treatment when compared with the 24-h experiment. After 24 h, 1,3,4-thiadiazoles produced little effects on the G0/G1 phase, and with the exception of **3c**, all produced a reduction in the S-phase population with a corresponding increase in G2 + M cell population. After 48 h of treatment, the compounds induced an arrest in the G2 + M phase, leading to an accumulation of cells in the G0/G1 phase and a reduction in the S-phase. The highest effect is produced by compound **3b**, while **3c** has the weakest one.

**Figure 9:**
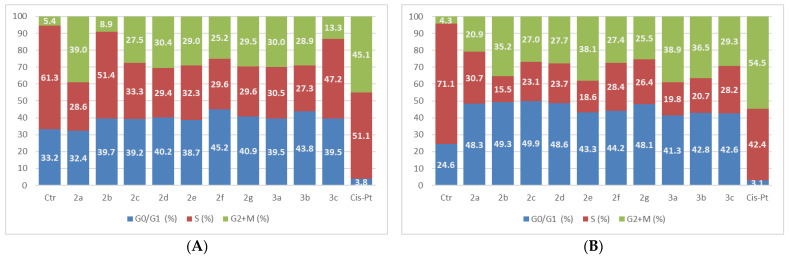
The tested compounds’ effects on cell cycle in LoVo cells. The cells were treated with the compounds and reference drug Cis-Pt at 50 µM for 24 h (**A**) and 48 h (**B**).

The compounds were also tested at a superior dose, in order to determine the effect on the cell cycle. The results of the compounds’ action on LoVo cells for the 50 µM dose are presented in Figure 9.

The treatment of LoVo cells with an elevated concentration of the compounds produced small changes in the cell phase distribution as compared with the results observed in the 5 µM experiments. The compounds elicited an augmentation in the G0/G1 phase accompanied by a reduction in the S phase and an elevation in the G2 + M population. This effect was more pronounced after a 48-h duration.

**Figure 10:**
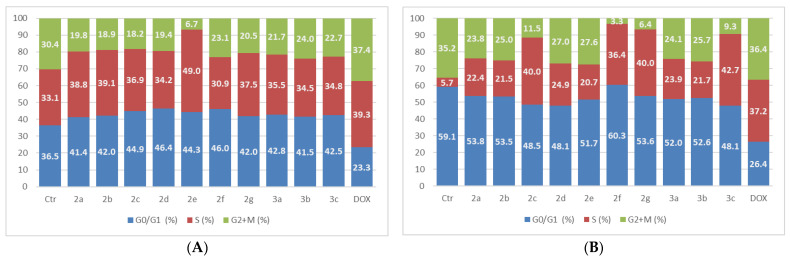
The tested compounds’ effects on cell cycle in MCF-7 cells. The cells were treated with the compounds and reference drug DOX at 5 µM for 24 h (**A**) and 48 h (**B**).

The compounds were tested on MCF-7 cells and the results for the 5 µM dose treatment are presented in Figure 10. DOX was used as a positive control and the experiments were performed after 24 h and 48 h of exposure.

The results indicate an elevated G0/G1 phase after 24 h that decreased after 48 h of treatment. The proportion of the S-phase cells increased in both experiments, while the number of cells in G2 + M is reduced compared with the control cells. The highest reduction in the G2 + M population was produced by compounds **2f** and **2g** after 48 h.

**Figure 11:**
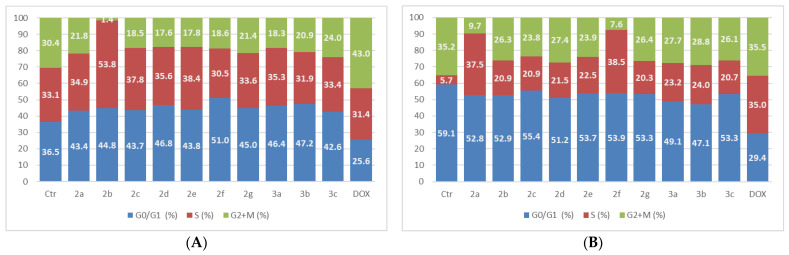
The tested compounds’ effects on cell cycle in MCF-7 cells. The cells were treated with the compounds and reference drug DOX at 50 µM for 24 h (**A**) and 48 h (**B**).

The results of the compounds’ action on MCF-7 cells for the 50 µM dose are presented in Figure 11.

After treating MCF-7 cells with 50 µM of thiadiazole derivatives for 24 h, there was an elevated proportion of cells in the G0/G1 phase; however, this trend reversed after 48 h. The percentage of cells in the S-phase showed an increase in both experiments, whereas the count of cells in G2 + M decreased compared to the control cells. The highest reduction in the G2 + M population was produced by compound **2b** after 24 h, and compound **2f** after 48 h.

### 2.3. Daphnia Magna Toxicity Assay

The *Daphnia magna* bioassay results are presented in Table 3 as the maximum average percentage of lethality (L%) registered for each compound and the median lethal concentration (LC_50_) if the values permitted its calculation. For each LC_50_ value calculated, the corresponding 95% confidence intervals (95%CI) are presented.

With the exception of **3a**, the tested thiadiazoles exhibited low lethality in the Daphnia assay after 24 h, with the average of the maximum lethality percentages remaining below the 50% threshold even at the highest concentration of 200 µM. The toxic effects were more pronounced in the 48-h experiment. Compound **3a** appears to be an outlier, inducing a substantial toxic effect that is significantly higher than that observed for all the other compounds tested.

### 2.4. Prediction of the Molecular Mechanism of Action

The PASS algorithm predicts a wide range of biological activities for a given molecule by analyzing its structural information. The output results are represented by two probabilities, Pa and Pi, for each specific target. The target profile for each compound was manually examined, and the pertinent oncotargets were chosen. The resulting Pa values for these selected targets are presented in Table 4.

The majority of targets predicted for the synthesized 1,3,4-thiadiazole compounds belong to the family of oncologic protein kinases. The analysis of the results for the new compounds **2f**,**g** and **3c** primarily indicates the inhibition of STAT transcription factors, particularly STAT3, and of the Mcl-1 protein as the most likely mechanisms responsible for the anti-proliferative effects. Compounds **2f**,**g** are predicted to also inhibit the cyclin-dependent kinase 9 (CDK9). These compounds have smaller predicted values as the unsubstituted derivative **2a**, but the Pa values serve as a measure of the likelihood of a compound interacting with a specific biological target rather than determining its potency. On the other hand, the amide derivative **3c** has a higher potential to inhibit STAT transcription factors compared to **2a**.

### 2.5. Molecular Docking

**Figure 12:**
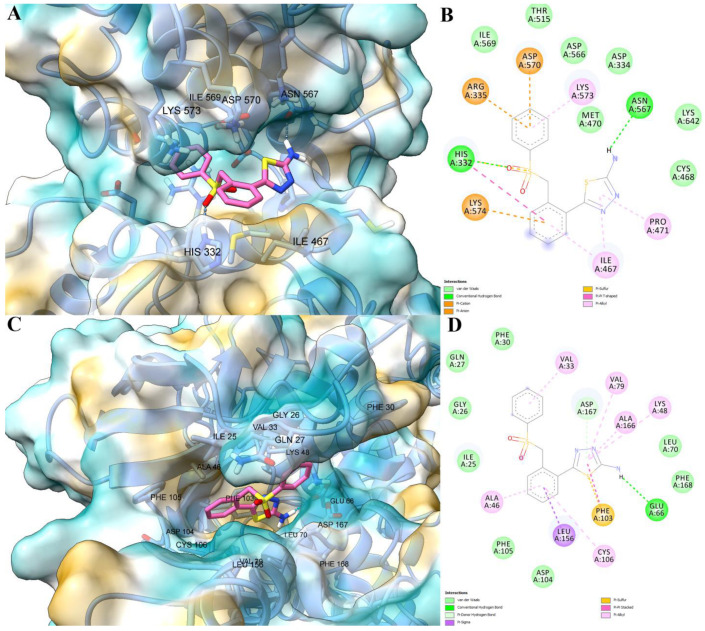
Predicted interactions between compound **2g** and potential targets STAT3 and CDK9. (**A**) Predicted conformation of compound **2g** in complex with STAT3 DBD, and blue dashed lines represent hydrogen bonds; (**B**) 2D diagram illustrating predicted interactions between compound **2g** and STAT3 DBD; (**C**) predicted conformation of compound **2g** in complex with CDK9 ATP-binding site, and blue dashed lines represent hydrogen bonds; (**D**) 2D diagram illustrating predicted interactions between compound **2g** and CDK9 ATP-binding site. Green dashed lines represent conventional hydrogen bonds, light green lines represent pi-donor hydrogen bonds, orange lines represent pi-cation/pi-anion interactions, purple lines represent pi-sigma interactions, magenta lines represent pi-pi stacked interactions, yellow lines represent pi-sulfur interactions, light pink lines represent pi-alkyl interactions.

The potential molecular mechanism of action was further investigated for compound **2g** by performing molecular docking simulations, since it exhibited the most promising antitumor effects. Based on PASS predictions, we selected transcription factor STAT3 and protein kinase CDK9 as target molecules, since both proteins are involved in tumorigenesis. STAT3 is structurally characterized by a coiled-coil domain, a DNA-binding domain (DBD), a linker domain, (LD), a Src homology 2 domain (SH2), and a transactivation domain (TD) [25]. Predictions based on the blind docking approach revealed that compound **2g** is most likely to inhibit STAT3 by interacting with the DNA-binding domain, similar to other validated STAT3 inhibitors (e.g., BBI-608 [26], inS3-54 [27]). Compound **2g** had a binding energy of −7.126 kcal/mol, for the binding site within STAT3 DBD. The novel compound formed a hydrogen bond with Asn567 via the amino group within the 2-amino-1,3,4-thiadiazol scaffold, while the sulfone moiety formed a hydrogen bond with His332. Furthermore, the 1,3,4-thiadiazole scaffold engaged in pi-alkyl interactions with Ile467 and Pro471. The two benzene rings formed hydrophobic interactions with several residues within the binding site, such as the pi-anion interaction with Asp570, pi-cation interactions with Arg335 and Lys574, pi-pi T-shaped interaction with His332 and pi-alkyl interactions with Lys573 and Ile467 (Figure 12A,B).

Secondly, we predicted the potential interaction between compound **2g** and the ATP-binding site of CDK9 (active conformation). Prior to investigating the novel compound, we first validated the docking protocol by redocking the co-crystallized inhibitor 5,6-dichloro-1-beta-D-ribofuranosyl-1H-benzimidazole (DRB). An RMSD value of 1.4740 Å was obtained after superposing the predicted pose on the experimental conformation, which is lower than the 2.0 Å threshold. The predicted binding energy for compound **2g** was −8.921 kcal/mol, which was lower than the predicted energy of the co-crystallized inhibitor (−7.481 kcal/mol). The docking experiment revealed that compound **2g** could form a hydrogen bond with the αC-helix Glu66 (αC_in_ conformation) through the amino group within the amino-thiadiazole scaffold. The 1,3,4-thiadiazole substructure formed a pi-sulfur interaction with the gate-keeper residue Phe103 via the sulfur atom, a pi-pi stacked interaction with the same residue, pi-alkyl interactions with Lys48 within the β3-strand, as well as with Val79 and Ala166, respectively, and a pi-donor hydrogen bond with Asp167. Moreover, the benzene rings also engaged in hydrophobic interactions, such as pi-alkyl interactions with Val33, Ala46 and Cys106 within the hinge region. Furthermore, a pi-sigma interaction is formed with Leu156 within the C-terminal lobe (Figure 12C,D).

## 3. Discussion

The objective of this research was the synthesis of new 1,3,4-thiadiazole derivatives and to evaluate their anticancer potential. The new compounds **2f**,**g** and **3c** share a central 5-phenyl-1,3,4-thiadiazol-2-amine scaffold and one or two additional benzene rings. The biological assays were performed on two cancer cell lines, LoVo and MCF-7.

LoVo is a human carcinoembryonic colon carcinoma cell line and represents an in vitro model for colon carcinoma [28]. The cells harbor a KRAS mutation (KRAS G13D mutant), but they present wild-type BRAF, PTEN, TP53 and PIK3CA [29]. Compared with the wild-type colon cancer cells, the G13D mutation on KRAS determines a reduction in the responsiveness of LoVo cells to cetuximab and panitumumab, drugs that target the epidermal growth factor receptor (EGFR) [30]. The MCF-7 cell line, derived from a pleural effusion at the Michigan Cancer Foundation, serves as a model for early-stage breast cancer characterized by functional estrogen receptor expression and dependence on estrogen for growth. Microarray analyses reveal that the gene set of MCF-7 clusters with the luminal subtype of breast cancer. MCF-7 harbors an E545K mutation in PIK3CA while maintaining wild-type TP53. The PIK3CA mutation is responsible for the increased activity of Akt [31].

Both compounds **2f**,**g** significantly reduced the viability of both LoVo and MCF-7 cells, with a higher impact after 48 h of incubation compared with the 24 h experiments, while compound **3c** had a low effect. The **2g** derivative had better anti-proliferative effects than **2f** in all experiments performed. The two compounds differ only by the sulfone group instead of the sulphur atom in the phenylthiomethyl fragment, highlighting its importance for the anti-proliferative effect. Comparing the anti-proliferative effect of **2g** with the other tested 5-aryl-1,3,4-thiadiazole-2-amine, it seems that the substitution in ortho with a hydrogen bonding group on the 5-phenyl fragment is important as highlighted by the effect of **2d** compared with **2c** or **2e**.

The best anti-proliferative effect on the LoVo cells was registered for **2g** after 48 h of exposure with an IC_50_ value of 2.44 µM, followed by **2d** with an IC_50_ value of 29.16 µM and **2a** with an IC_50_ value of 62.16 µM. The same order of potency was observed on MCF-7 cells, but the IC_50_ values were higher and **2c** had a better effect than **2a**. The differences between the effects on the two cell lines are observed also in the modifications of the cell cycle. After 48 h, compound **2g** produced an augmentation in the G0/G1 phase and in the G2 + M population accompanied by a reduction in the S phase in LoVo cells; however, in the MCF-7 cells, an increase in the S-phase was observed, while the count of cells in G2 + M phase decreased. The compound exhibited low toxicity in the *Daphnia* test, with lethality percentages below 20%, even at the highest concentration of 200 µM. 

The PASS predictive studies indicate the inhibition of STAT transcription factors, Mcl-1 protein and CDK9 as the most likely mechanisms responsible for anti-proliferative effects. All of these targets are known to be involved in cancer cells’ proliferation. STAT are a family of transcription factors that function as cytoplasmic regulators of gene transcription by integrating various signaling pathways [32]. Several inhibitors of STAT3 determined potent anti-proliferative effects when tested against LoVo and various other human colon cancer cell lines [33,34,35]. Mcl-1 belongs to the anti-apoptotic subset of BCL-2 family proteins, playing a role in modulating the equilibrium between cellular survival and death; its pharmacologic blockade produces a reduction in cell proliferation [36]. CDK9 plays a crucial role in the initiation and advancement of various cancers, contributing significantly to tumorigenesis, as its inhibition is a promising strategy against cancer [37,38]. A series of 2-amino-5-pyrimidine-thiazole derivatives, chemically similar to compound **2g**, demonstrated potent anti-proliferative effects on MCF-7 cells and on HCT-116 colon carcinoma, as well as by inhibiting CDK9 [39]. The molecular docking studies highlighted that compound **2g** can potentially inhibit STAT3 transcriptional activity by interfering with DNA binding, and CDK9 kinase activity by interacting with the active conformation of the ATP-binding domain. The docking study underlines the importance of the sulphone and amino groups for the inhibition of STAT3, in agreement with the higher effect of **2g** compared to **2f** or with **3a**. 

The **3a**–**c** compounds can be considered derivatives of **2a**. The transformation of **2a** in **3a**,**c** was significantly detrimental for the anti-proliferative effect on both cell lines tested. In the case of **3b**, the effect on MCF-7 was similar to that produced by **2a**, but it was less active on LoVo cells. The IC_50_ registered for **3b** on MCF-7 after 48 h is higher than that of 5.87 µM, as reported by Yang et al. [16], probably because of the differences in the assay protocol. Compounds **3a**–**c** also had significant toxic effects in the *Daphnia* assay, especially **3a**, the acetyl derivative of **2a**.

The new compounds **2f**,**g** and **3c** increased the cellular apoptosis in both LoVo and MCF-7 cells in a similar manner, with a higher impact after 48 h of incubation compared with the 24 h experiments, but with little differences between the 5 µM and 50 µM doses. The effect on apoptosis is consistent with the predicted mechanisms of action [40,41]. The diminished impact of the compounds on LoVo cells in contrast to their effects on MCF-7 cells can be explained by the intrinsic resistance of the LoVo cells stemming from inactivating mutations in BAX, a pro-apoptotic member of the Bcl-2 protein family [42].

The study demonstrated the potential of the newly synthesized thiadiazole derivatives for anticancer drug development, based on their anti-proliferative effects and modulation of apoptosis, and of the cell cycle dynamics in LoVo and MCF-7 cells. 

## 4. Materials and Methods

### 4.1. Analytical Procedures

The melting points (m.p.) were determined using an Electrothermal 9100 apparatus (Bibby Scientific Ltd., Stone, UK) in open capillary tubes and are reported without any corrections. The ^1^H-NMR and ^13^C-NMR spectra were acquired on a Mercury 300 BB instrument (Varian Medical Systems, Inc., Palo Alto, CA, USA) operating at frequencies of 300 MHz for ^1^H and 75.075 MHz for ^13^C and on a Bruker Avance III (Karlruhe, Germany) operating at 500 MHz for ^1^H and 125 MHz for ^13^C. Chemical shifts were recorded as δ values in ppm units, referenced to tetramethylsilane (TMS) used as the internal standard. DMSO-d6 was employed as solvent. The coupling constants (J) were reported in hertz (Hz), and the splitting patterns were abbreviated as follows: s, singlet; d, doublet; t, triplet; q, quartet; and b, broad. The infrared (IR) spectra were obtained on a FT/IR-4200 spectrometer (JASCO, Tokyo, Japan) equipped with an ATR PRO450-S accessory. Elemental analyses were performed on a Perkin-Elmer 2400 Series II CHNS/O Elemental Analyzer (Shelton, CT, USA).

### 4.2. Synthesis Procedures

All the chemicals and reagents were purchased from commercial suppliers and used without purification, unless otherwise noted.

**Scheme 1:**
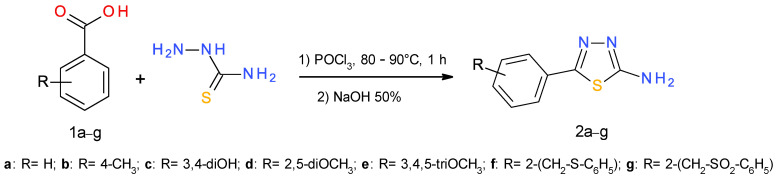
Synthesis of 1,3,4-thiadiazole-2-amine derivatives (**2a**–**g**).

The 1,3,4-thiadiazole-2-amine derivatives (**2a**–**g**) were prepared by cyclodehydration of aromatic carboxylic acids (**1a**–**g**) with thiosemicarbazide in the presence of phosphorus oxychloride. Among these compounds, **2f**,**g** are reported for the first time. The method was adapted based on previously described procedures [43,44] and the reaction is presented in Scheme 1. The aromatic carboxylic acids **1f**,**g** were prepared starting from thiophenol and phtalide, according to previously reported procedures [45,46,47].

**Scheme 2:**
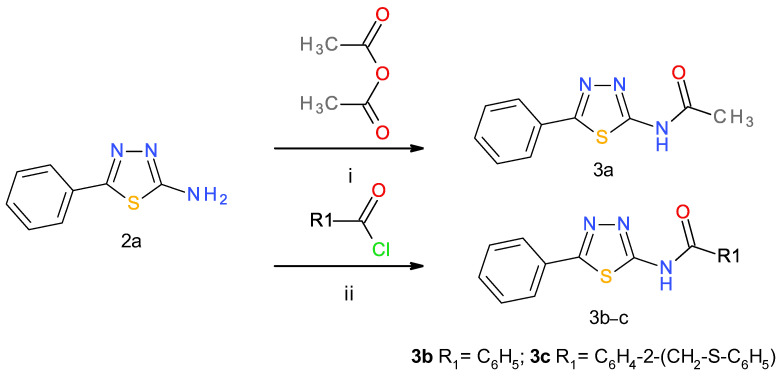
Synthesis of 1,3,4-thiadiazol-2-amide derivatives. **3**. Reagents and conditions: (**i**) pyridine, reflux, and 2 h; (**ii**) anhydrous THF, 24 h, and r.t.

The 1,3,4-thiadiazol-2-amide derivatives **3a**–**c** were synthesized from 5-phenyl-1,3,4-thiadiazol-2-amine (**2a**). Compound **3a** was obtained by acetylation of **2a** with acetic anhydride, while compounds **3b**–**c** were obtained through the coupling reaction between **2a** and the corresponding acid chlorides in anhydrous tetrahydrofuran (THF). Solid sodium hydrogenocarbonate was used for the neutralization of liberated hydrogen chloride. Among these compounds, **3c** is reported here for the first time. The synthesis methods were adapted based on previously described procedures [43,48] and are presented in Scheme 2.

The acid chloride required for the synthesis of **3c** (2-(phenylthiomethyl)benzoic acid chloride) was prepared in situ by refluxing 2-(phenylthiomethyl)benzoic acid with thionyl chloride in 1,2-dichloroethane, followed by solvent evaporation under reduced pressure.

**Scheme 3:**
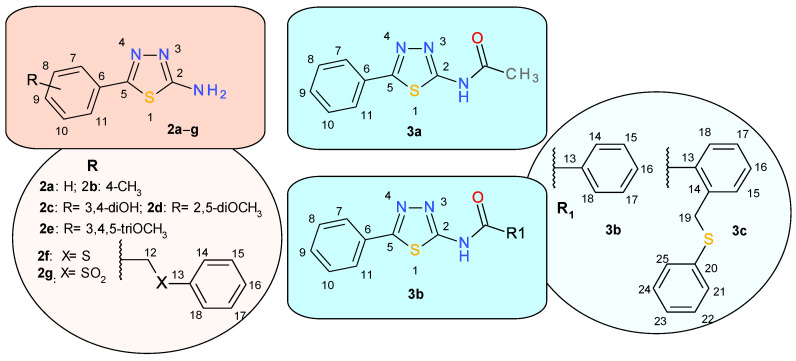
The numbering of atoms in the 1,3,4-thiadiazole derivatives **2a**–**g** and **3a**–**c**.

Scheme 3 presents the atoms’ numbering used for assigning the NMR signals of the new thiadiazole derivatives.

#### 4.2.1. General Procedure for the Synthesis of 5-Aryl-1,3,4-thiadiazole-2-amine Derivatives (**2a**–**g**)

A mixture of acid **1a**–**g** (3.00 mmol) and POCl_3_ (10 mL) was stirred for 20 min at room temperature. Then, thiosemicarbazide (3.00 mmol) was added and the resulting mixture was heated at 80–90 °C for one hour, under stirring. The reaction mixture was cooled in an ice bath, 40 mL of water was added carefully and the resulting suspension was refluxed for 4 h. The mixture was cooled followed by a basification of the solution to pH 8 using a 50% sodium hydroxide solution, and it was stirred. The solid obtained was filtered and recrystallized from an appropriate solvent.

#### 4.2.2. 5-Phenyl-1,3,4-thiadiazol-2-amine (**2a**)

This is a white solid with a yield of 91% and an m.p. of 227–228 °C. ^1^H-NMR (500 MHz, DMSO, δ ppm, *J* Hz): 7.75 (d, 2H, H-7, H-11); 7.41–7.47 (m, 5H, H-8, H-9, H-10, NH2). 13C-NMR (125 MHz, DMSO, δ ppm): 168.51 (C-2); 156.46 (C-5); 131.04 (C-6); 129.62 (C-9); 129.17 (C-8, C-10); 126.35 (C-7, C-11). FT-IR (ATR in solid, ν cm^−1^): 3268; 3061; 2959; 2774; 1633; 1509; 1468; 1334; 1264; 1138; 1058; 758; 686; 496; 437. The elemental analysis calculated for C_8_H_7_N_3_S (177.23 g/mol) is: C 54.22%, H 3.98%, N 23.71% and S 18.09%; it is found to be: C 54.61%, H 3.26%, N 24.10% and S 17.92%.

#### 4.2.3. 5-(4-Methylphenyl)-1,3,4-thiadiazol-2-amine (**2b**)

This is a white solid with a yield of 89% and an m.p. of 214–215 °C. ^1^H-NMR (500 MHz, DMSO-d6, δ ppm, *J* Hz): 7.61 (d, 8.0, 2H, H-8, H-10); 7.35 (s, 2H, NH_2_, deuterable); 7.26 (d, 8.0, 2H, H-7, H-11); 2.33 (s, 3H, CH_3_). ^13^C-NMR (125 MHz, DMSO-d6, δ ppm): 168.15 (C-2); 156.45 (C-5); 139.25 (C-9); 129.61 (C-8, C-10 C-7, C-11); 128.30 (C-6); 126.22 (C-7, C-11); 20.85 (CH_3_). FT-IR (ATR in solid, ν cm^−1^): 3268; 3082; 2954; 1628; 1507; 1465; 1325; 1256; 1178; 1129; 1052; 977; 811; 688. The elemental analysis calculated for C_9_H_9_N_3_S (191.26 g/mol) is: C 56.52%, H 4.74%, N 21.97% and S 16.76%; it is found to be: C 56.22%, H 5.02%, N 21.41% and S 16.98%.

#### 4.2.4. 5-(3,4-Dihydroxyphenyl)-1,3,4-thiadiazol-2-amine (**2c**)

This is a beige solid with a yield of 62% and an m.p. of 315–316 °C. ^1^H-NMR (300 MHz, DMSO-d6, δ ppm, *J* Hz): 9.37 (bs, 1H, HO); 9.29 (bs, 1H, HO); 7.18 (bs, 2H, NH_2_); 7.19 (d, 2.0, 1H, H-7); 6.98 (dd, 2.0, 8.2, 1H, H-11); 6.78(d, 8.2, 1H, H-10). ^13^C-NMR (75 MHz, DMSO-d6, δ ppm): 167.89 (C-2); 157.39 (C-5); 147.61 (C-9); 146.04 (C-8); 122.85 (C-6); 118.87 (C-11); 116.38 (C-10); 113.62 (C-7). FT-IR (ATR in solid, ν cm^−1^): 3418; 3298; 3193; 3035; 1610; 1497; 1439; 1377; 1338; 1295; 1261; 1218; 1163; 1114; 1047; 917; 881; 801; 754; 640; 546. The elemental analysis calculated for C_8_H_7_N_3_O_2_S (209.23 g/mol) is: C 45.93%, H 3.37%, N 20.08% and S 15.32%; it is found to be: C 46.20%, H 3.01%, N 20.59% and S 14.91%.

#### 4.2.5. 5-(2,5-Dimethoxyphenyl)-1,3,4-thiadiazol-2-amine (**2d**)

This is a white solid with a yield of 61% and an m.p. of 188–189 °C. ^1^H-NMR (300 MHz, DMSO-d6, δ ppm, *J* Hz): 7.62 (d, 2.5, 1H, H-11); 7.22 (bs, 2H, NH_2_); 7.13 (d, 8.8, 1H, H-8); 7.00 (dd, 2.5, 8.8, 1H, H-9); 3.86 (s, 3H, OCH_3_); 3.75 (s, 3H, OCH_3_). ^13^C-NMR (75 MHz, DMSO-d6, δ ppm): 170.29 (C-2); 153.50 (C-5); 150.58 (C-10); 149.12 (C-7); 120.33 (C-6); 116.97 (C-9); 114.24 (C-11); 110.58 (C-8); 56.73 (C-12 or C-13); 55.63 (C-12 or C-13). FT-IR (ATR in solid, ν cm^−1^): 3417; 3296; 3191; 3034; 1609; 1497; 1437; 1378; 1337; 1293; 1260; 1218; 1162; 1113; 1047; 917; 881; 801; 753; 640; 545. The elemental analysis calculated for C_10_H_11_N_3_O_2_S (237.28 g/mol) is: C 50.62%, H 4.67%, N 17.71% and S 13.51%; it is found to be: C 50.98%, H 4.18%, N 17.97% and S 13.20%.

#### 4.2.6. 5-(3,4,5-Trimethoxyphenyl)-1,3,4-thiadiazol-2-amine (**2e**)

This is a white solid with a yield of 75% and an m.p. of 226–227 °C. ^1^H-NMR (500 MHz, DMSO-d6, δ ppm, *J* Hz): 7.39 (s, 2H, NH_2_); 7.00 (s, 2H, H-7, H-11); 3.84 (s, 6H, OMe-8, OMe-10); 3.70 (s, 3H, OMe-9). ^13^C-NMR (125 MHz, DMSO-d6, δ ppm): 168.48 (C-2); 156.21 (C-5); 153.20 (C-8, C-10); 138.67 (C-9); 126.49 (C-6); 103.69 (C-7, C-11); 60.11 (OMe-9); 56.02 (OMe-8, OMe-10). FT-IR (ATR in solid, ν cm^−1^): 3341; 3269; 3031; 2937; 2831; 1585; 1500; 1456; 1401; 1321; 1232; 1117; 1066; 989; 800; 743. The elemental analysis calculated for C_11_H_13_N_3_O_3_S (267.31 g/mol) is: C 49.43%, H 4.90%, N 15.72% and S 12.00%; it is found to be: C 49.02%, H 4.62%, N 16.08% and S 12.44%.

#### 4.2.7. 5-[2-(Phenylthiomethyl)phenyl]-1,3,4-thiadiazol-2-amine (**2f**)

This is a white solid with a yield of 60% and an m.p. of 139–140 °C. ^1^H-NMR (500 MHz, DMSO-d6, δ ppm, *J* Hz): 7.78 (bs, 2H, NH_2_); 7.53 (m, 1H, H-11); 7.41 (m, 1H, H-9); 7.38–7.34 (m, 2H, H-8, H-10); 7.31–7.23 (m, 4H, H-14, H-18 and H-15, H-17); 7.18 (m, 1H, H-16); 4.56 (s, 2H, H-12). ^13^C-NMR (125MHz, DMSO-d6, δ ppm): 169.07 (C-2); 155.21 (C-5); 135.91 (C-13); 135.70 (C-7); 129.29 (C-6); 131.08 (CH-9); 130.56 (CH-11); 129.44 (C-8 or C-10); 128.95 (C-14, C-18 and C-15, C-17); 127.88 (C-8 or C-10); 126.15 (C-16); 35.59 (C-12). FT-IR (ATR in solid, ν cm^−1^): 3303; 3122; 1651; 1619; 1506; 1436; 1247; 1076; 1034; 982; 744; 696; 680; 584. The elemental analysis calculated for C_15_H_13_N_3_S_2_ (299.42 g/mol) is: C 60.17%, H 4.38%, N 14.03% and S 21.42%; it is found to be: C 60.52%, H 4.03%, N 14.52% and S 21.01%.

#### 4.2.8. 5-[2-(Benzenesulfonylmethyl)phenyl]-1,3,4-thiadiazol-2-amine (**2g**)

This is a white solid with a yield of 59% and an m.p. of 238–239 °C. ^1^H-NMR (500 MHz, DMSO-d6, δ ppm, *J* Hz): 7.66 (tt, 1.7, 7.4, 1H, H-16); 7.48 (bt, 7.6, 2H, H-15, H-17); 7.46–7.35 (m, 6H, H-8, H-9, H-10, H-11, H-14, H-18); 5.37 (s, 2H, H-12). ^13^C-NMR (125MHz, DMSO-d6, δ ppm): 168.88 (C-2); 155.94 (C-5); 138.36 (C-13); 130.88 (C-6); 126.18 (C-7); 133.73 (C-16); 133.56 (C-8, C-9 or C-10); 130.42 (C-8, C-9 or C-10); 129.35 (C-8, C-9 or C-10); 129.00 (C-15, C-17); 128.85 (C-11); 127.78 (C-14, C-18); 57.39 (C-12). FT-IR (ATR in solid, ν cm^−1^): 3409; 3259; 3125; 3001; 2942; 1593; 1501; 1303; 1144; 1082; 987; 763; 728; 687; 609; 515. The elemental analysis calculated for C_15_H_13_N_3_O_2_S_2_ (331.42 g/mol) is: C 54.36%, H 3.95%, N 12.68% and S 19.35%; it is found to be: C 54.05%, H 4.20%, N 12.19% and S 19.62%.

#### 4.2.9. Synthesis of N-(5-Phenyl-1,3,4-thiadiazol-2-yl)acetamide (**3a**)

A mixture of 5-phenyl-1,3,4-thiadiazol-2-amine **2a** (1.8 mmol), acetic anhydride (3.6 mL) and pyridine (3 mL) was refluxed for 2 h. After the solution was cooled, the resulting crystals were collected by filtration and recrystallized from methanol.

This is a white solid with a yield of 83% and an m.p. of 275–276 °C. ^1^H-NMR (500 MHz, DMSO-d6, δ ppm, *J* Hz): 12.64 (s, 1H, NH); 7.94 (m, 2H, H-7, H-11); 7.54–7.51 (m, 3H, H-8, H-9, H-10); 2.22 (s, 3H, H-13). ^13^C-NMR (125 MHz, DMSO-d6, δ ppm): 168.77 (C-12); 161.75 (C-2); 158.47 (C-5); 130.31 (C-6); 130.51 (C-9); 129.43 (C-8, C-10); 126.98 (C-7, C-11); 20.53 (C-13). FT-IR (ATR in solid, ν cm^−1^): 3369; 3154; 3039; 2768; 1686; 1552; 1433; 1370; 1308; 1236; 1115; 965; 846; 757; 716; 688. The elemental analysis calculated for C_10_H_9_N_3_OS (219.27 g/mol) is: C 54.78%, H 4.14%, N 19.16% and S 14.62%; it is found to be: 54.98%, H 3.97%, N 19.42% and S 14.31%.

#### 4.2.10. General Procedure for the Synthesis of 1,3,4-thiadiazol-2-amide Derivatives (**3b**,**c**)

To the solution of the corresponding acid chloride (2.50 mmol) in dry tetrahydrofuran (25 mL), 5-phenyl-1,3,4-thiadiazol-2-amine **2a** (2.00 mmol) and NaHCO_3_ (10 mmol) were added, and the resulting mixture was stirred for 24 h at room temperature. Afterwards, the solvent was evaporated under reduced pressure, and 40 mL of water was added to the residue, as well as solid Na_2_CO_3_ until pH 9.5 was reached; the formed suspension was stirred for 1 h at the room temperature and filtered. The final compounds were recrystallized from aqueous solution of 70% ethanol.

#### 4.2.11. N-(5-Phenyl-1,3,4-thiadiazol-2-yl)benzamide (**3b**)

This is a white solid with a yield of 88% and an m.p. of 237 °C. ^1^H-NMR (300 MHz, DMSO-d6, δ ppm, *J* Hz): 13.16 (bs, 1H, NH); 8.12 (dd, 1.2, 8.2, 2H, H-7, H-11); 7.98 (m, 2H, H-14, H-18); 7.68 (tt, 1H, H-9, 1.4, 8.2); 7.61–7.50 (m, 5H, H-8, H-10, H-15, H-16, H-17). ^13^C-NMR (75 MHz, DMSO-d6, δ ppm): 165.22 (C-12); 162.11 (C-2 or C-5); 159.37 (C-5 or C-2); 131.47 (C-6); 130.19 (C-13); 133.08 (C-16); 130.66 (C-9); 129.40 (C-8, C-10); 128.70 (C-15, C-17); 128.44 (C-14, C-18); 126.96 (C-7, C-11). FT-IR (ATR in solid, ν cm^−1^): 3108; 3063; 2995; 2901; 2768; 1666; 1524; 1438; 1304; 1254; 1072; 1025; 888; 800; 761; 626. The elemental analysis calculated for C_15_H_11_N_3_OS (281.34 g/mol) is: C 64.04%, H 3.94%, N 14.94% and S 11.40%; it is found to be: C 63.87%, H 4.20%, N 15.02% and S 11.10%.

#### 4.2.12. 2-Phenylthiomethyl-N-(5-phenyl-1,3,4-thiadiazol-2-yl)benzamide (**3c**)

This is a white solid with a yield of 72% and an m.p. of 204–205 °C. ^1^H-NMR (300 MHz, DMSO-d6, δ ppm, *J* Hz): 13.18 (bs, 1H, NH); 8.00 (m, 2H, H-7, H-11); 7.70 (bd, 7.0, 1H, H-18); 7.58–7.52 (m, 3H, H-arom); 7.47–7.38 (m, 3H, H-arom); 7.30–7.20 (m, 4H, H-21, H-25, H-22, H-24); 7.14 (tt, 1H, H-23, 1.7, 7.2); 4.49 (s, 2H, H-19). ^13^C-NMR (75 MHz, DMSO-d6, δ ppm): 167.20 (C-12); 162.12 (C-2 or C-5); 158.82 (C-5 or C-2); 137.38 (C-14); 135.58 (C-20); 132.79 (C-13 or C-6); 130.20 (C-6 or C-13); 131.14 (CH); 130.65 (CH); 129.13 (CH); 127.28 (CH); 126.30 (C-23); 130.65 (C-9); 129.40 (C-8, C-10); 129.15 (C-21/C-25 or C-22/C-24); 128.93 (C-22/C-24 or C-21/C-25); 126.96 (C-7, C-11); 34.97 (C-19). FT-IR (ATR in solid, ν cm^−1^): 3117; 3064; 2996; 2906; 2775; 1669; 1574; 1525; 1476; 1432; 1297; 1250; 1057; 1033; 884; 805; 763; 723; 678. The elemental analysis calculated for C_22_H_17_N_3_OS_2_ (403.53 g/mol) is: C 65.48%, H 4.25%, N 10.41% and S 15.89%; it is found to be: C 65.69%, H 4.01%, N 10.04% and S 16.11%.

### 4.3. Anticancer Evaluation

#### 4.3.1. Reagents

Doxorubicin (DOX), Cisplatin (Cis-Pt), dimethyl sulfoxide (DMSO), trypsin-EDTA (0.25% trypsin and 0.03% EDTA), Glutamine (Glu), Dulbecco’s modified Eagle’s medium (DMEM), fetal bovine serum (FBS), Propidium Iodide (PI) (stock solution of 4 mg/mL PI in PBS) and RNase A (stock solution of 10 mg/mL RNase A) were purchased from Sigma–Aldrich (St. Louis, MO, USA). Annexin V-FITC kit and CycleTEST PLUS DNA Reagent kit was purchased from Becton Dickinson Biosciences, San Jose, CA, USA. The stock solutions were prepared by dissolving the compounds in a minimum amount of DMSO and kept at −20 °C. The working solutions were prepared from the stocks with the culture medium before each experiment.

#### 4.3.2. Cell Culture and Treatments

LoVo (human colorectal adenocarcinoma) human cancer cell line and HUVEC normal cells were purchased from American Type Culture Collection (ATCC). The MCF-7 human breast adenocarcinoma cell line was provided from European Collection of Authenticated Cell Cultures (ECACC). HUVEC normal cells, isolated from the vascular endothelium of an umbilical cord, were used as a reference. Adherent cells were maintained in culture in Dulbecco’s modified Eagle medium/nutrient mixture F-12 (DMEM:F12) with 2 mM of L-glutamine, 10% fetal bovine serum, 100 units/mL of penicillin and 100 μg/mL of streptomycin, and incubated at 37 °C in a 5% CO_2_-humidified atmosphere. After 24 h, adherent cells were treated with different concentrations of the compounds for different periods of time. Cis-Pt and DOX, conventional oncology drugs used in cancer treatments, can be used as a positive control of the experiments. Cell treatments of compounds, Cis-Pt and DOX, were carried out using concentrations of 400, 200, 100, 50, 25, 12.5 and 6.25 μM of the tested compound. Then, cells were detached with a nonenzymatic solution of phosphate-buffered saline (PBS)/1 mM EDTA and washed twice in PBS.

#### 4.3.3. Cytotoxicity Assay

The study utilized a colorimetric assay based on MTS (chemically known as 3-(4,5-dimethylthiazol-2-yl)-5-(3-carboxymethoxyphenyl)-2-(4-sulfophenyl)-2*H*-tetrazolium) and called CellTiter 96 Aqueous One Solution Cell proliferation Assay (Promega, Madison, WI, USA) in order to measure cell viability. The assay was performed in triplicate in 96-well microtiter plates with a flat bottom (Falcon). A number of 1 × 10^4^ cells/well were cultured in 100 μL for 24 h, culture supernatants were discarded, and then cells were treated for 24 and 48 h with increasing concentrations of the tested compound. After the end of the incubation time, 20 μL of the reagent containing MTS and PES (phenazine ethosulfate) was added in each well. Plates were incubated for 4 h at 37 °C, with mild agitation every 15 min. The reduction in the tetrazolium compound to formazan was spectrophotometrically measured at λ = 492 nm using a Dynex plate reader (DYNEX Technologies MRS, Chantilly, VA, USA). The percentage of viability compared to untreated cells (considered 100% viable) was calculated with the formula:

(1) $$Cell\;viability\;(\%)=\frac{Absorbance\;treated\;cells-Absorbance\;culture\;medium}{Absorbance\;untreated\;cells-Absorbance\;culture\;medium}\times 100$$

The cell viability compared was calculated and data were expressed as a mean ± standard deviation (SD) of the experiments and obtained in triplicate (n = 3) [49].

#### 4.3.4. Apoptosis Analysis

The apoptosis assay was performed according to the manufacturer’s instructions using the Annexin V-FITC kit (BD Biosciences, San Jose, CA, USA). Cells untreated and treated with the experimental compound were suspended in a cold binding buffer. The percentages of apoptotic cells were determined by double staining with Annexin V-FITC/PI. In each tube was added 400 μL of Annexin V binding buffer and the 5000 cells/sample for 15 min in the dark at room temperature. The cells were analyzed using a FACSCantoII flow-cytometer (Becton Dickinson—BD, La Jolla, CA, USA). The analysis was performed using DIVA 6.2 software to discriminate viable cells (FITC-PI-) from necrotic cells (FITC + PI+), and early apoptosis (FITC + PI) from late apoptosis [50,51].

#### 4.3.5. Cell Cycle Analysis

In this experiment, 5 × 10^5^ cells that had been previously fixed were washed and suspended in PBS. The CycleTEST PLUS DNA Reagent kit from BD Biosciences was used according to the manufacturer’s protocol to carry out the assay. The probes were kept in the dark and at 4 °C until data acquisition by flow-cytometry (Becton Dickinson—BD) using a FACSCantoII flow-cytometer. The ModFIT software (https://www.vsh.com/products/mflt/index.asp, accessed on 11 December 2023) was used to analyze the data and estimate the DNA index (DI) and progression through cell-cycle phases [52].

### 4.4. Daphnia Magna Toxicity Assay

*Daphnia magna* Straus is parthenogenetically maintained at ‘Carol Davila’ University’s Department of Pharmaceutical Botany and Cell Biology. The culture was consistently maintained at a temperature of 25 °C, with a 16-h light and 8-h dark photoperiod. To ensure consistency, young daphnids were carefully chosen based on their size and kept in an artificial medium for 24 h prior to the assessment. The bioassay involved 10 daphnids per replicate, using tissue culture plates with 12 wells from Greiner Bio-One. The experimental procedure followed the protocol described in previous studies [53,54].

Each determination was carried out in duplicate. The median lethal concentration (LC_50_) and their corresponding 95% confidence intervals (95%CI) were calculated using the least square fit method in the GraphPad Prism v 5.1 software.

### 4.5. Prediction of the Molecular Mechanism of Action

A virtual screening was conducted using the PASS (Prediction of Activity Spectra for Substances) application. This algorithm is specifically designed to assess the general biological activity of compounds based on their structure [55]. The input for the screening consisted of mol files of the compounds, and the results were analyzed based on whether the output probability to be active (Pa) was higher than the probability to be inactive (Pi). All the pharmaceutical targets identified were then manually filtered based on their potential for an anti-cancer treatment [56].

### 4.6. Molecular Docking

A molecular docking study was performed to predict the potential interactions between the novel compound with the most promising antitumor activity and key molecular targets involved in tumorigenesis. Based on PASS results, we selected the transcription factor STAT3 (signal transducer and activator of transcription 3) and CDK9 (cyclin-dependent kinase 9) for docking simulations. Crystal structures of the mouse STAT3 monomer in a complex with DNA and human CDK9 in a complex with activator cyclin-T1 and inhibitor DRB (5,6-dichloro-1-beta-D-ribofuranosyl-1H-benzimidazole) were retrieved from the RCSB PDB database (PDB IDs: 1BG1, 2.25 Å resolution [57], and 3MY1, 2.8 Å resolution [58]). Protein structures were optimized for docking using the YASARA structure [59] by removing water molecules, correcting structural errors, protonation according to the physiological pH (7.4), and optimizing the hydrogen-bonding network, followed by energy minimization with the YASARA2 forcefield. 

Ligands were prepared by generating 3D structures with DataWarrior 5.2.1 [60]. Ligand structures were energetically minimized using semi-empirical quantum mechanics (MOPAC) and protonated according to the physiological pH. The AutoDock Vina v1.1.2 algorithm [61] was used for carrying out the simulations, performing 12 runs for each target. The grid box was set to include the ATP-binding site of CDK9 and the entire structure of STAT3. We used the full-length structure of STAT3 for defining the search space (blind docking), since multiple binding sites were reported for known inhibitors. The docking protocol was validated for CDK9/Cyclin-T1 by redocking the co-crystallized inhibitor, followed by the superposition of the predicted pose with the experimental coordinates and calculation of the RMSD (root mean square deviation) value. The docking results were retrieved as predicted binding poses and binding energies (ΔG, kcal/mol). The predicted molecular interactions between the ligand and target proteins were analyzed using UCSF ChimeraX v1.6.1 [62] and BIOVIA Discovery Studio Visualizer (BIOVIA, Discovery Studio Visualizer, Version 17.2.0, Dassault Systèmes, 2016, San Diego, CA, USA)

## 5. Conclusions

The synthesis of thiadiazole derivatives presented in this study demonstrates their potential as candidates for anticancer drug development. The observed anti-proliferative effects, induction of apoptosis, and blockade of cell cycle dynamics in both LoVo and MCF-7 cells highlight the importance of the 5-phenyl-1,3,4-thiadiazol-2-amine scaffold, as well as the specific additional substitution groups. The incorporation of the sulfonyl group as a substituent onto the phenyl moiety in close proximity to the thiadiazole ring while keeping the amino group free seems to be a promising avenue for future development. The novel compound **2g**, 5-[2-(benzenesulfonylmethyl)phenyl]-1,3,4-thiadiazol-2-amine, emerged as the lead for future structure optimization studies based on its higher anti-proliferative effects and lower toxic effects in *Daphnia* experiments, as compared with the other compounds tested here. PASS predictions and molecular docking studies supported the hypothesis that compound **2g** could act as a STAT3 and CDK9 inhibitor. Future studies should also focus on elucidating the underlying molecular mechanisms to further validate the therapeutic potential of this compound.

## Figures and Tables

**Table 1:** The calculated IC_50_ values on the three cell lines after exposure for 24 h and 48 h.

Substance	IC_50_ (24 h)			IC_50_ (48 h)		
	HUVEC	LoVo	MCF-7	HUVEC	LoVo	MCF-7
**2a**	>400	203.60	>400	83.77	62.16	>400
**2b**	>400	133.70	>400	70.92	124.5	>400
**2c**	>400	163.3	>400	155.10	117.40	397.80
**2d**	>400	53.64	154.30	94.80	29.16	121.50
**2e**	>400	107.60	>400	300.30	79.19	>400
**2f**	>400	>400	>400	>400	80.70	>400
**2g**	>400	18.08	43.34	44.55	2.44	23.29
**3a**	>400	>400	>400	>400	>400	>400
**3b**	>400	174.10	>400	>400	62.53	>400
**3c**	>400	>400	>400	>400	>400	>400
Cis-Pt	11.14	12.84	NT *	8.23	80.13	NT *
DOX	1.03	NT *	129.10	1.35	NT *	87.39

* NT = not tested.

**Table 2:** The results of the apoptosis assay on HUVEC, LoVo and MCF-7 cells.

Substance	Conc.	Total Apoptosis (%) 24 h			Total Apoptosis (%) 48 h		
		HUVEC	LoVo	MCF-7	HUVEC	LoVo	MCF-7
Control	-	9.4	5.2	6.1	11.2	8.7	10.3
**2a**	5 μM	12.8	9.0	11.8	14.7	17.6	24.4
	50 μM	15.0	8.9	9.8	18.4	13.7	24.7
**2b**	5 μM	16.7	6.9	8.0	15.7	14.0	16.7
	50 μM	13.6	6.3	8.4	16.6	12.3	25.4
**2c**	5 μM	16.6	7.5	11.7	15.6	13.0	26.6
	50 μM	12.1	6.8	7.6	12.1	13.8	15.1
**2d**	5 μM	28.4	13.5	14.6	14.4	19.3	37.6
	50 μM	19.8	12.0	11.8	16.8	17.9	34.9
**2e**	5 μM	26.3	4.9	9.1	13.1	14.3	24.6
	50 μM	24.3	16.4	14.9	15.5	19.9	34.2
**2f**	5 μM	19.9	14.9	22.0	15.9	27.0	44.0
	50 μM	18.6	8.2	12.6	14.6	17.4	45.0
**2g**	5 μM	19.0	13.6	18.7	14.7	23.6	42.2
	50 μM	19.3	19.6	19.7	14.1	24.8	44.5
**3a**	5 μM	26.6	14.9	17.6	18.2	20.1	24.9
	50 μM	22.6	14.5	17.9	20.1	22.3	47.9
**3b**	5 μM	17.8	12.5	19.2	15.3	22.2	43.7
	50 μM	17.0	12.6	16.4	15.9	20.9	41.3
**3c**	5 μM	15.5	14.8	16.4	13.5	27.6	39.3
	50 μM	16.9	11.2	14.3	14.7	24.5	37.3
Cis-Pt	5 μM	15.7	15.7	-	14.8	31.6	-
	50 μM	21.7	24.5	-	17.4	38.7	-
DOX	5 μM	15.2	-	14.8	9.5	-	33.6
	50 μM	18.6	-	19.0	10.6	-	49.0

**Table 3:** Daphnia magna bioassay: 24 h and 48 h values; 95% confidence intervals for newly synthetized compounds and their starting materials.

Compound	24 h			48 h		
	Maximum Average L%	LC50 (µM)	95%CI of LC50 (µM)	Maximum Average L%	LC50 (µM)	95%CI of LC50 (µM)
**2a**	0	ND	-	40	ND	-
**2b**	10	ND	-	55	143.60	21.0–980.0
**2c**	0	ND	-	25	ND	-
**2d**	40	240.80	138.7–418.1	95	50.66	31.7–80.9
**2e**	0	ND	-	5	ND	-
**2f**	5	ND	-	10	ND	-
**2g**	0	ND	-	20	ND	-
**3a**	95	<6.25	-	100	<6.25	-
**3b**	5	ND	-	45	228.30	93.53–557.1
**3c**	10	ND	-	55	188.5	69.59–510.4

ND not determined due the results obtained.

**Table 4:** The probability of compounds **2a**–**g** and **3a**–**c** to be active (Pa) as predicted by PASS analysis.

Target	2a	2b	2c	2d	2e	2f	2g	3a	3b	3c
Transcription factor STAT inhibitor	0.45	0.48	0.39	0.37	0.37	0.47	0.23	0.67	0.73	0.64
Transcription factor STAT3 inhibitor	0.44	0.48	0.39	0.38	0.38	0.35	–	0.70	0.74	0.67
Mcl-1 antagonist	0.44	0.40	0.44	0.32	0.37	0.43	0.31	0.49	0.55	0.46
Kinase inhibitor	–	–	0.38	–	–	–	–	–	–	–
Protein kinase AGC family inhibitor	0.23	0.17	0.18	0.13	0.15	0.09	–	0.22	0.24	0.14
Protein kinase B inhibitor	0.37	0.30	0.24	0.18	0.23	0.14	0.11	0.25	0.15	0.08
Protein kinase B alpha inhibitor	0.32	0.28	0.30	0.26	0.26	0.19	0.14	0.17	0.23	–
Cyclin-dependent kinase inhibitor	0.31	0.19	0.14	0.10	0.13	0.08	0.09	0.47	0.30	0.13
Cyclin-dependent kinase 2 inhibitor	0.29	0.18	0.12	0.12	0.12	0.09	0.08	0.41	0.26	0.12
CDK9/cyclin T1 inhibitor	0.47	0.43	0.46	0.36	0.35	0.36	0.32	–	0.24	–
Pfmrk inhibitor	0.20	0.20	0.16	–	–	–	–	0.16	0.23	–
MAP3K5 inhibitor	0.23	–	–	–	–	0.25	–	0.35	0.35	0.30
MAP kinase kinase 4 inhibitor	0.27	0.23	0.31	0.25	0.23	–	–	–	0.21	–
p21-activated kinase 1 inhibitor	0.20	–	0.22	0.20	–	–	–	–	0.19	–
Protein-tyrosine kinase (PTK) inhibitor	0.32	0.26	0.25	0.37	0.24	0.13	0.15	0.18	0.28	0.14
Abl kinase inhibitor	0.31	0.26	0.24	0.37	0.23	0.13	0.14	0.18	0.28	0.13
Tyrosine-protein kinase Fgr inhibitor	–	–	0.20	–	–	–	–	–	0.44	0.30
Protein-tyrosine kinase p55 (blk) inhibitor	–	–	–	–	–	–	–	–	0.22	–
Focal adhesion kinase inhibitor	0.20	0.18	–	0.20	0.22	–	–	0.21	0.28	0.19
Focal adhesion kinase 2 inhibitor	0.28	0.27	0.22	0.24	0.29	–	–	0.27	0.37	0.27
Platelet-derived growth factor receptor kinase inhibitor	–	0.26	–	–	–	–	–	–	0.37	–
Proteasome ATPase inhibitor	–	–	–	–	–	–	–	0.47	–	–

## Data Availability

Data are contained within the article or Supplementary Materials.

## Supplementary Materials

The supporting information can be downloaded at: https://www.mdpi.com/article/10.3390/ijms242417476/s1.
