# Supplementary material for: Synthesis of 1,3,4-Thiadiazole Derivatives and Their Anticancer Evaluation
Source: Int J Mol Sci. 2023 Dec 14;24(24):17476. doi: 10.3390/ijms242417476 (PMC10743895; doi:10.3390/ijms242417476)
Supplement: Supplementary file 1 [file ijms-24-17476-s001.zip › ijms-2760882-supplementary.pdf]

Supplementary Materials

Synthesis of 1,3,4-Thiadiazole Derivatives and their Anticancer Evaluation

Camelia Elena Stecoza [1], George Mihai Nitulescu [1,*], Constantin Draghici [2], Miron Teodor Caproiu [2], Anamaria Hanganu [2], Octavian Tudorel Olaru [1], Dragos Paul Mihai [1],Marinela Bostan [3] and Mirela Mihaila [3]

Table S1. The 95% confidence intervals for the calculated IC_{50} values.

Substance	Confidence intervals (24 h)			Confidence intervals (48 h)		
	**HUVEC**	**LoVo**	**MCF-7**	**HUVEC**	**LoVo**	**MCF-7**
2a	NC *	147.4 to 281.2	NC *	75.57 to 92.85	55.16 to 70.05	NC *
2b	NC *	109.7 to 163.1	NC *	44.10 to 114.1	92.19 to 168.2	NC *
2c	NC *	135.5 to 196.8	NC *	134.8 to 178.4	95.09 to 144.9	294.6 to 537.3
2d	NC *	43.92 to 65.51	126.2 to 188.6	73.14 to 122.9	26.01 to 32.69	113.5 to 130.2
2e	NC *	88.12 to 131.3	NC *	199.6 to 451.6	57.24 to 109.5	NC *
2f	NC *	NC *	NC *	NC *	67.22 to 96.90	NC *
2g	NC *	16.01 to 20.41	38.98 to 48.20	35.71 to 55.57	1.62 to 3.66	20.39 to 26.59
3a	NC *	NC *	NC *	NC *	> 400	NC *
3b	NC *	156.6 to 193.5	NC *	NC *	47.13 to 82.95	NC *
3c	NC *	NC *	NC *	NC *	> 400	NC *
Cis-Pt	8.93 to 13.89	10.19 to 16.18	NT*	6.71 to 10.09	69.62 to 92.23	NT*
DOX	0.52 to 2.04	NC *	94.57 to 176.3	0.96 to 1.90	NC*	73.45 to 104.0

* NC = not calculated.
